# Influence of Iron Regulation on the Metabolome of *Cryptococcus neoformans*

**DOI:** 10.1371/journal.pone.0041654

**Published:** 2012-07-23

**Authors:** Jung Nam Choi, Jeongmi Kim, Jiyoung Kim, Won Hee Jung, Choong Hwan Lee

**Affiliations:** 1 Department of Bioscience and Biotechnology, Konkuk University, Seoul, Republic of Korea; 2 Department of Biotechnology, Chung-Ang University, Anseong-Si, Gyeonggi-Do, Republic of Korea; Research Institute for Children and the Louisiana State University Health Sciences Center, United States of America

## Abstract

Iron is an essential nutrient for virtually all organisms and acts as a cofactor for many key enzymes of major metabolic pathways. Furthermore, iron plays a critical role in pathogen-host interactions. In this study, we analyzed metabolomic changes associated with iron availability and the iron regulatory protein Cir1 in a human fungal pathogen *Cryptococcus neoformans*. Our metabolite analysis revealed that Cir1 influences the glycolytic pathway, ergosterol biosynthesis and inositol metabolism, which require numerous iron-dependent enzymes and play important roles in pathogenesis and antifungal sensitivity of the fungus. Moreover, we demonstrated that increased cellular iron content and altered gene expression in the *cir1* mutant contributed to metabolite changes. Our study provides a new insight into iron regulation and the role of Cir1 in metabolome of *C. neoformans*.

## Introduction

Iron is an essential nutrient for virtually all organisms and plays as a cofactor for many key enzymes of major metabolic pathways, such as the tricarboxylic acid cycle, respiration, amino acid biosynthesis, and the synthesis of lipids and sterols [1]. Acquisition of iron from the environment is problematic, mainly because of its insoluble nature under aerobic conditions. To overcome extremely low bioavailability of iron, organisms have developed various efficient iron transport systems. Examples include reduction of ferric iron to ferrous iron to increase its permeability and secretion of iron chelating molecules siderophores [2]. Although its essential roles, iron causes detrimental effects if exceed. Free iron catalyzes the formation of oxygen radicals via the Fenton reaction, which causes protein denaturation, DNA breakage, etc. Therefore, iron acquisition and homeostasis are tightly regulated in a cell.

Iron also plays a critical role in pathogen-host interactions. Typically, both the pathogen and the host require iron and compete over it during the infection process. While the hosts have developed various means to limit the availability of intra- or intercellular iron contents upon infection, the pathogens have evolved distinct systems for survival within the host environment. Therefore the mechanisms of iron transport and homeostasis in pathogens have been extensively studied [3].

*Cryptococcus neoformans* is a fungal pathogen and a leading cause of pulmonary and central nervous systemic mycosis in immunocompromised individuals such as HIV-infected patients. *C. neoformans* has been considered an excellent model fungal pathogen to study iron transport and homeostasis because of its intriguing connection with virulence. For example, iron deprivation influences the formation of the polysaccharide capsule and synthesis of melanin, which are considered major virulence factors [4]. Moreover, recent studies suggested that the high-affinity reductive iron transport pathway is required for full virulence in a mouse model of cryptococcosis and for proliferation in brain tissue [5], [6]. A study aiming to identify the major iron regulatory protein in *C. neoformans* revealed that a GATA-type zinc finger transcription factor Cir1 controls expression of genes involved in iron transport and homeostasis [4]. The same study also showed that expression of genes required for major virulence factors such as melanin formation and capsule synthesis were regulated by Cir1. Furthermore, a number of genes required for signaling and metabolic pathways were shown to be influenced by deletion of *CIR1* suggesting importance of the iron regulatory protein in physiology of *C. neoformans*. Finally, the strains lacking *CIR1* became avirulent suggesting significance of the protein in pathogenesis of *C. neoformans* [4]. Although the previous study suggested that Cir1 plays a major regulatory role in transcriptional networks related to iron metabolism and virulence, the metabolic consequences of deletion of *CIR1* are largely unknown. Therefore, we applied metabolite analysis to further understand the functions of Cir1 in *C. neoformans*.

Metabolomics profiles cellular metabolites and is a rapidly expanding area strategy in the post-genomics era [7]. In metabolite profiling, it is preferable to use a wide-spectrum metabolite analysis technique, which is rapid, reproducible and stable during sample analysis [8]. Recent technological advances in mass spectrometry have realized reliable and highly sensitive measurements of metabolites [9]. Among them, gas chromatography mass spectrometry (GC–MS) has been successfully applied to analyze and interpret multiparametric metabolic responses in living systems to pathopysiological and environmental perturbations [10]. In the last few decades, a number of chemometric tools have been applied to the interpretation and quality assessment of MS-based metabolomic data. Recently, an analysis of metabolite profiles was performed to understand how iron deficiency affected the metabolomes of a common laboratory strain *Saccharomyces cerevisiae,* which revealed that iron deficiency led to changes in glucose metabolism, amino acid biosynthesis, and lipid biosynthesis [11]. In the current study, we aimed to identify specific metabolic pathway that is mostly influenced by iron or Cir1 and to understand the role of Cir1 in the metabolome of *C. neoformans*. Our data showed that abundance of some of metabolites, such as those involved in the glycolytic pathway, ergosterol biosynthesis or inositol metabolism, was changed in the *cir1* mutant.

**Figure 1:**
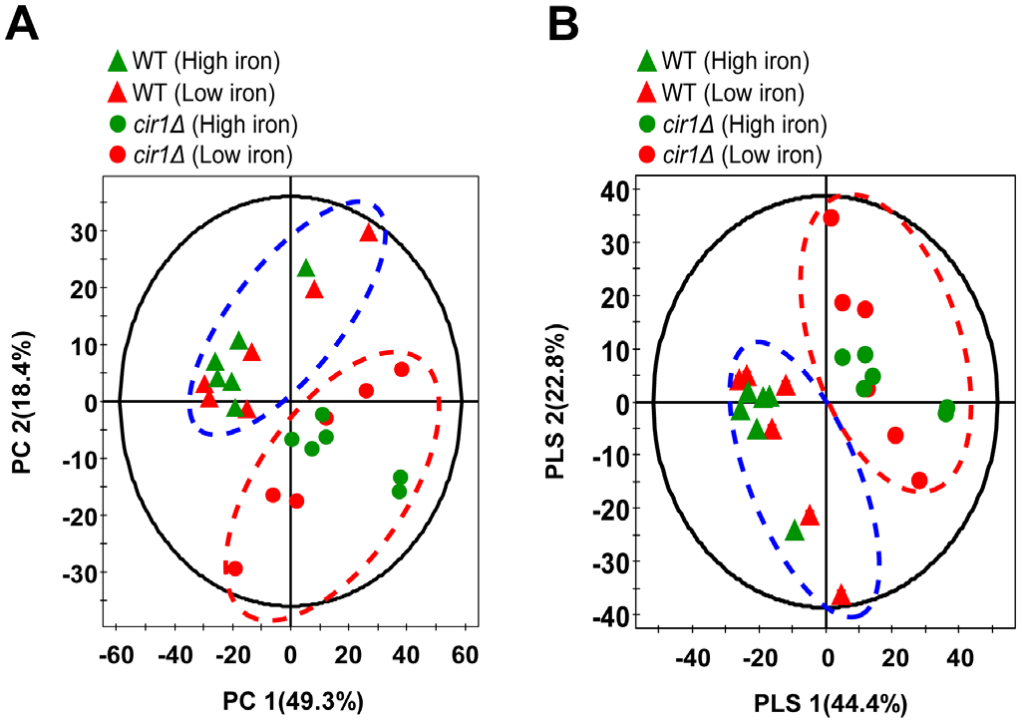
The principal component analysis (PCA) score scatter plot (A) and partial least square-discriminate analysis (PLS-DA) score scatter plot (B) represent significant differences of metabolites between the wild-type (WT) and the *cir1* mutant (*cir1Δ*). Data show difference of 24 samples (6 biological replicates of the wild-type or the cir1 mutant grown in low- or high-iron medium). Remarkable score plots were observed in clusters at the given strain, respectively. However, no statistically significant difference was observed when metabolites of the strains grown in low-iron medium were compared with that of the strains grown in high-iron medium.

## Materials and Methods

### Strains and Growth Conditions

The strains used in this study were routinely grown in YPD (1% yeast extract, 2.0% bacto-peptone, 2.0% glucose), YNB (yeast nitrogen base, Difco) with 2.0% glucose. Defined low-iron media containing 5 g of glucose, 5 g of asparagine, 400 mg of K_2_HPO_4_, 80 mg of MgSO_4_•7H_2_O, 250 mg of CaCl_2_•2H_2_O, 57 µg of boric acid, 5 µg of CuSO_4_•5H_2_O, 10 µg of MnCl_2_•4H_2_O, 2 mg of ZnSO_4_•7H_2_O, and 4.6 µg of sodium molybdate per liter. Low-iron water was prepared using Chelex-100 (Bio-Rad) resin. The strains used in this study were *C. neoformans* var. *neoformans* serotype D B3501A (the wild-type) and B3CIR572 (the *cir1* mutant), which were constructed previously [4]. To extract metabolites, cells were pre-grown in 50 ml of low-iron YNB overnight at 30°C. After incubation, cells were collected by centrifugation at 4,000 rpm for 5 min, were washed twice with low-iron water, and were resuspended in 25 ml of defined low-iron media. Suspension of cells was diluted 1/10 in 50 ml of defined low-iron media, followed by incubating at the same temperature for 12 h with shaking. Cells were transferred to 150 ml of fresh defined low-iron media with or without 100 mM FeCl_3_, grown for additional 12 h, and were harvested. Cell pellets were used for metabolomic analysis. Total six independent cultures (biological replicates) for each strain were prepared for each growth condition and were subsequently analyzed throughout the study.

**Figure 2:**
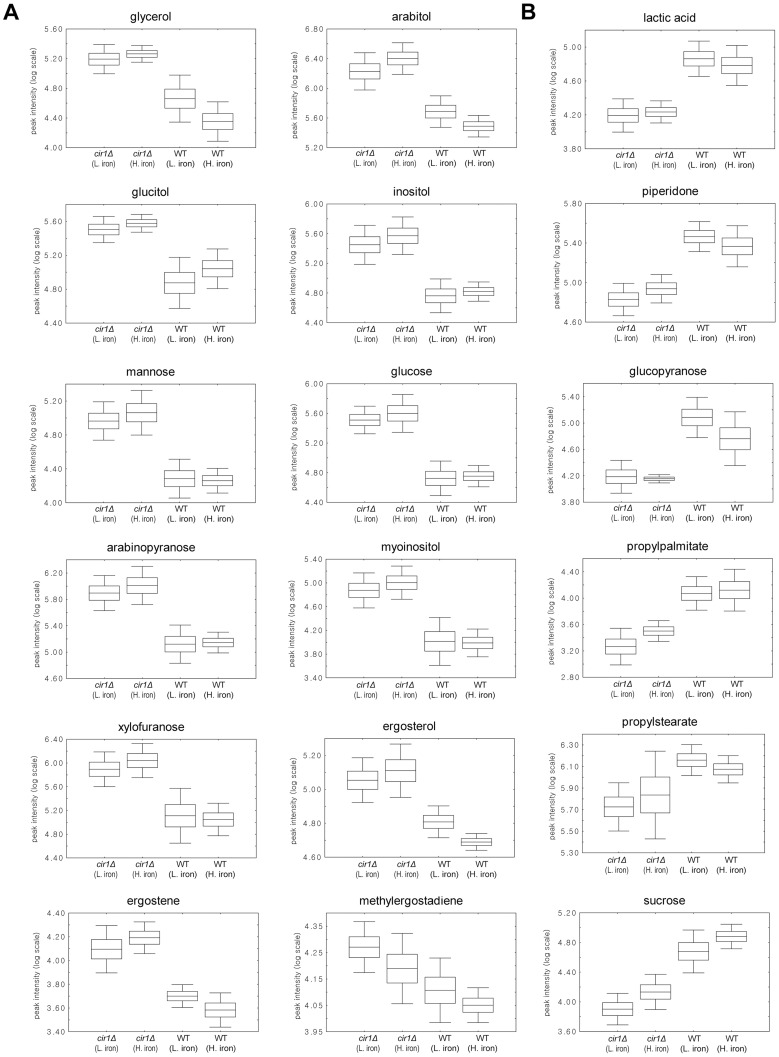
Box-and-whisker plots illustrating discriminative metabolites of the *cir1* mutant (*cir1Δ*) and the wild-type (WT) grown in low- (L.iron) or high-iron (H.iron) medium. A total of 18 discriminative metabolites were identified in the *cir1* mutant. Among them, 12 metabolites were increased in the *cir1* mutant (A), and 6 were decreased compared to that of the wild-type (B).

**Table 1:** The most significantly different metabolites identified in the strain lacking CIR1 relative to the wild-type.

Compound	Rt^a^ (min)	Fragment Pattern (*m/z*)	*p*-value^b^	derivatized^c^
lactic acid	6.27	219, 191, 147, 133, 117, 88, 73	6.59E-5	TMSi (x2)
glycerol	9.32	293, 218, 205, 147, 133, 117, 103, 89, 73	2.46E-7	TMSi (x3)
piperidone	11.96	258, 243, 201, 169, 147, 128, 115, 100, 73	1.95E-6	TMSi (x2)
arabitol	14.86	395, 319, 307, 277, 243, 205, 147, 129, 117, 103, 73	2.98E-5	TMSi (x5)
glucopyranose	15.79	435, 393, 305, 231, 217, 204, 191, 147, 129, 103, 73	3.64E-4	TMSi (x5)
glucitol	17.13	421, 319, 307, 277, 217, 205, 147, 117, 103, 73	2.04E-7	TMSi (x6)
inositol	17.35	612, 507, 318, 305, 217, 191, 171, 147, 129, 73	7.17E-5	TMSi (x6)
mannose	17.50	435, 393, 305, 231, 217, 204, 191, 147, 129, 117, 73	1.14E-4	TMSi (x4)
glucose	17.56	435, 379, 305, 217, 204, 191, 147, 103, 73	3.53E-5	TMSi (x5)
arabinopyranose	17.89	332, 305, 217, 204, 191, 147, 129, 73	4.54E-4	TMSi (x4)
myoinositol	18.01	507, 432, 318, 305, 265, 217, 191, 147, 129, 73	6.08E-4	TMSi (x6)
xylofuranose	18.29	376, 318, 305, 217, 147, 103, 73	7.86E-4	TMSi (x4)
propyl palmitate	22.12	459, 371, 313, 239, 203, 147, 129, 103, 73, 57	5.00E-4	TMSi (x2)
sucrose	22.43	569, 361, 331, 228, 217, 204, 169, 147, 129, 103, 73	4.73E-6	TMSi (x8)
propyl stearate	23.52	487, 399, 267, 217, 203, 147, 129, 95, 73	3.77E-3	TMSi (x2)
ergosterol	27.07	468, 378, 363, 338, 293, 253, 211, 157, 73	1.62E-5	TMSi (x1)
ergostene	27.86	473, 457, 368, 318, 255, 229, 213, 159, 147, 133, 105, 75	2.74E-6	TMSi (x1)
methyl-ergostadiene	28.37	469, 495, 380, 357, 296, 267, 227, 200, 173, 147, 121, 105, 73	1.58E-3	TMSi (x1)

a Rt, retention time.

b Level of significance (*p*-value <0.005) of the difference between strains tested.

c Number of hydrogen atoms derivatized.

### Preparation of Fungal Extracts

In order to separate broth and the cells, harvested culture broths were centrifuged at 1,500 rpm. Collected cells were subjected to glass bead lysis and metabolites were extracted with 75% boiling ethanol. Extracts were dried in a freezing dryer (12 h) which were derivatized in two steps to protect carbonyl functions. First, the dried samples were dissolved in 100 µl of 20 mg/ml solution of methoxyamine hydrochloride in pyridine (Sigma) and incubated at 75°C for 30 min. The volatility of polar compounds was increased by exchanging acidic protons against trimethysilyl group using 100 µl of *N*-methyl-*N*-trimethylsilyltrifluoroacetamide (MSTFA) (Sigma) at 70°C for 30 min.

**Table 2:** Total cellular iron contents in the strains.

Strain	Low-iron	High-iron
WT	0.40±0.03*	0.38±0.01
*cir1Δ*	0.76±0.05	0.99±0.07

* Iron concentration (mole/g) of the cells ± standard deviation. Values were calculated from three independent experiments and normalized with total protein concentration.

### GC-MS Based Non-targeted Metabolite Profiling

Each 1 µl of the derivatives was injected in a split mode (1∶25) into a Varian CP-3800 gas chromatography system coupled to a Varian 4000 ion-trap EI/CI mass spectrometric detector system (Varian, CA). The samples were analyzed using a VF-1MS capillary column (30 m×0.25 mm) coated with 0.25-µm low bleed polymer, a FactorFour™ column (Varian), equipped with an integrated 10 m guard column (Varian, CA, USA), and vaporized to separate the derivative metabolites at 250°C. For analysis, the initial oven temperature was held at 70°C for 2 min; ramped to 300°C at a rate of 10°C /min and held for 5 min. Helium (purity >9.999%) was used as a carrier gas at a constant flow rate of 1 ml/min. The temperature of the EI ion source and injector were set to 200°C and 250°C, respectively. The electron impact ionization (70 eV) was utilized, and mass data were collected in a full-scan mode (*m/z* 50–1000). The metabolites were identified by comparison to the NIST 2005 database (version 2.0, FairCom Co., USA).

**Figure 3:**
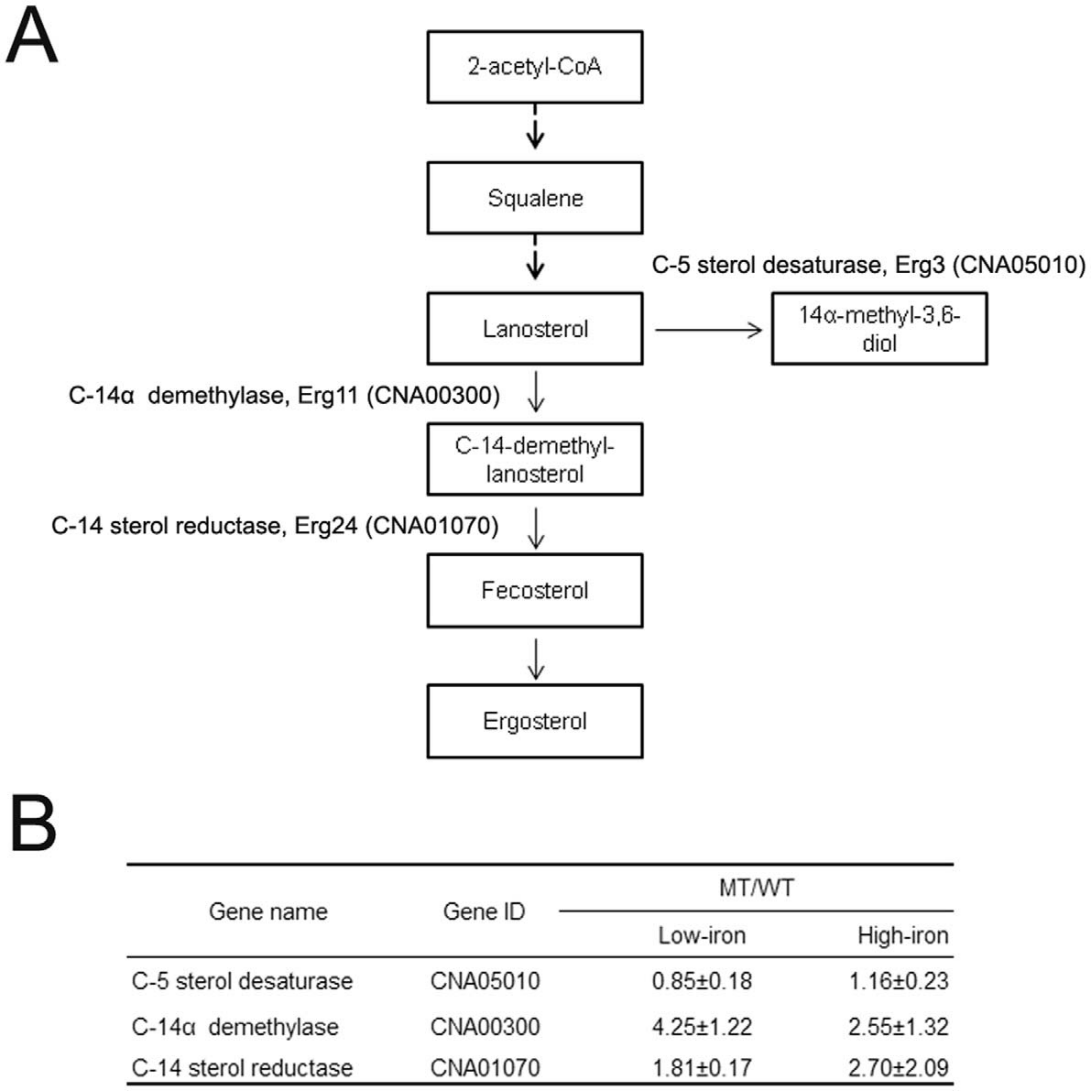
Summary of the ergosterol synthesis pathway in *C. neoformans* (A). The *cir1* mutant displayed increased levels of ergosterol and its derivatives. Up-regulation of the genes, *ERG11* and *ERG24*, contributed altered ergosterol contents in the *cir1* mutant (B).

**Figure 4:**
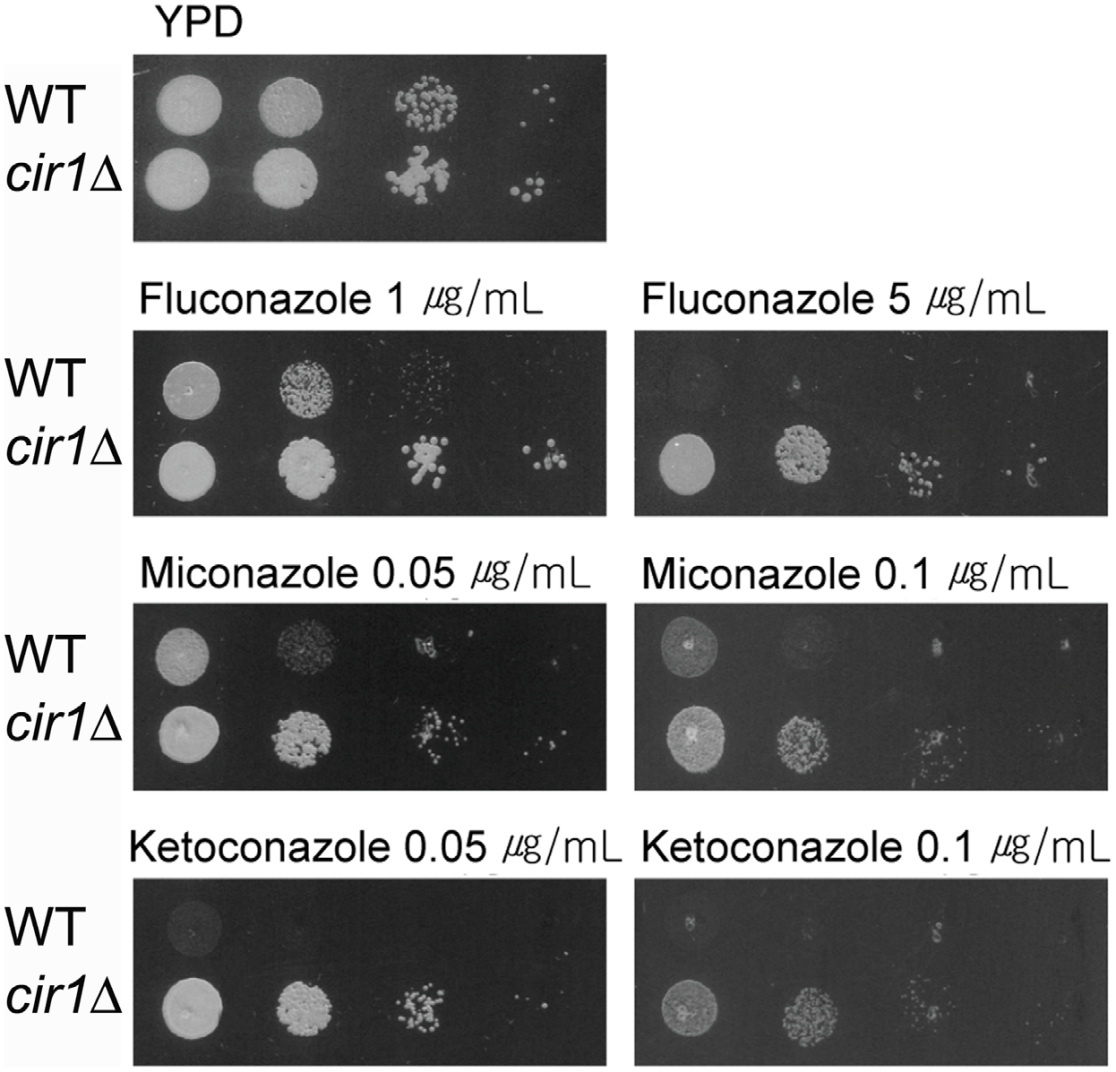
The *cir1* mutant displayed decreased sensitivity to azole antifungal drugs. The growth of the wild-type (WT) or the *cir1* mutant (*cir1Δ*) in media containing an antifungal drug was monitored. Ten-fold serial dilutions of cells (starting at 10^4^ cells) were spotted onto YPD plates with the drug indicated. Plates were incubated at 30°C for 2 days.

### Spectral Data Processing

The raw GC-MS data were converted into CDF (NetCDF) files by using the Vx capture software (version 2.1, Laporte, MN) and subsequently processed by XCMS toolbox for automatic peak detection and alignment. R-program version 2.9.0 (www.r-project.org) and XCMS version 1.16.3 were used. The XCMS parameters for the R language were performed by simple commands as XCMS’s default settings (http://masspec.scripps.edu/xcms/documentation.php). For multivariate statistical analysis, the XCMS output was further processed using Microsoft Excel (Microsoft, Redmond, WA, USA). The data were arranged on a three-dimensional matrix consisting of arbitrary peak index (RT-*m/z* pair), sample names (observations), and peak area (variables).

**Figure 5:**
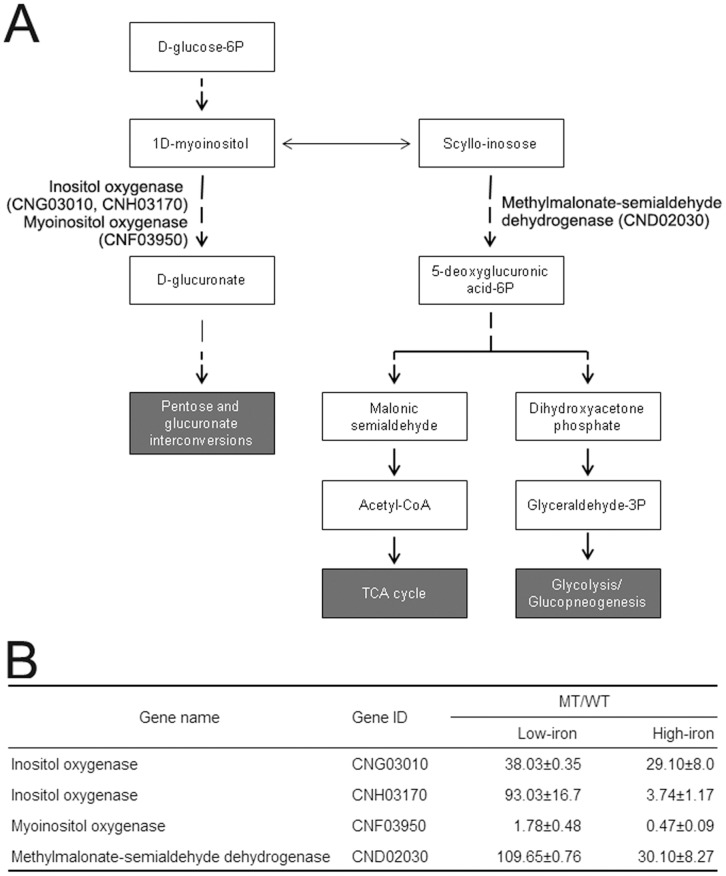
Summary of the inositol metabolic pathway in *C. neoformans* (A). The *cir1* mutants displayed increased levels of inositol. The up-regulated genes in the *cir1* mutant are listed in the table (B).

### Multivariate Statistical Analysis

The resulting three-dimensional data table was entered into the SIMCA-P^+^ (version 12.0, Umetrics, Umeå, Sweden) software package for multivariate statistical analysis. Unsupervised principal component analysis (PCA) was performed to observe general clustering and trends of metabolite differences among all samples. Supervised partial least squares discriminant analysis (PLS-DA) model was used to maximize metabolite variations and identify significantly altered metabolites responsible for such variations. [12] The quality of the fitting model can be explained by *R^2^X* and *Q^2^Y* values. *R^2^X* display the variance explained in the model and indicates the goodness of fit. *Q^2^Y* display the cumulative predicted variation in *Y*. The values of these parameters approaching 1.0 indicated a stable model with a predictive reliability. The VIP (variable importance in the projection) values were calculated and used to identify the most significantly different metabolites for clustering of specific class-separating and were further validated by Student’s *t*-test using STATISTICA (version 7.0, StatSoft Inc., Tulsa, OK). Metabolites with VIP values greater than 1.0 and *p* values less than 0.05 (threshold) were selected as discriminating metabolites between the wild-type and the mutant strains. Box and whisker plots were generated in STATISTICA to show the significantly difference of chemistry measurement between the 2 classes of samples. Pair-wise metabolite-to-metabolite correlations were calculated using the Pearson's correlation coefficient (*rMet*) test using the R programs.

### Determination of Cellular Iron Concentration

To determine iron concentration, the wild-type strain and the *cir1* mutants were grown as describe above. Cell cultures were washed three times with low-iron water and mixed with lysis buffer containing 50 mM HEPES-KOH pH 7.5, 140 mM NaCl, 1 mM EDTA and 1% Triton ×−100. Cells were disrupted and homogenized using glass-beads, and iron concentration of lysate was measured by using QuantiChrom™ Iron Assay Kit (Bioassay Systems, DIFE-250) according to the manufacturer’s instruction. Briefly, 50 µl of cell lysate (7.5 µg of total protein) was mixed with 200 µl of the working reagent mixture and incubated at room temperature for 40 min. Optical density of the sample at 590 nm was measured and iron concentration was calculated with a standard curve generated from iron standard solutions.

### Phenotypic Analysis

To estimate antifungal drug sensitivity, the wild-type and the *cir1* mutant strain were grown in YPD overnight at 30°C with shaking. After incubation, 10-fold dilutions of cells (starting at 10^4^ cells) were spotted onto YPD plates containing various azole antifungal drugs. The plates were incubated at 30°C for 2 days and photographed.

### Quantitative Real-time RT-PCR

Primers for real-time RT-PCR were designed using Primer Express software 3.0 (Applied Biosystems) and are listed in Table S1. Cell cultures prepared as described above. Total RNA was extracted using the RiboPure™-Yeast Kit (Ambion), and cDNA was synthesized using the RevertAid™ First Strand cDNA synthesis Kit (Fermentas). Relative gene expression was quantified using 7500 software v.2.0.3 (Applied Biosystems) based on the 2^−ΔΔC^_T_ method. 18S rRNA was used as endogenous control.

## Results and Discussion

### Non-targeted Metabolite Profiling of *C. neoformans* by Using MS

The current study focused on determining the influence of Cir1 on the metabolome in *C. neoformans*. A total of 6 biological replicates, cultured in the absence or presence of an inorganic iron source (FeCl_3_) were prepared for the wild-type and the mutant strain lacking *CIR1*, and their metabolites were analyzed using MS based non-targeted metabolite profiling. In general, ion chromatogram does not provide an evidence of difference and similarity of complex sample sets and, therefore, often requires further sophisticated data analysis. We chose XCMS software for GC-EI-MS/MS data processing, and applied a normalization method to the total spectral area to obtain aligned metabolome data of the wild-type and the *cir1* mutants individually [13], [14], [15]. The processed metabolite profiles (972 peaks, extracted by XCMS from the GC-EI-MS/MS data sets) were used to compare metabolite profiles of the strains in each growth condition via multivariate statistical analysis (Table S2).

Because of the qualitative similarity and complexity of the metabolome data sets, visualization of preliminary metabolite changes is challenging; thus, chemometric multivariate statistical analysis was carried out using principal component analysis (PCA) [16]. Data in the score plot of mass spectra showed clear separation, with good cross-validation, between the wild-type and the *cir1* mutant. Moreover, we evaluated the quality of our models by using *R^2^X* and *Q* ^2^ values, which indicate goodness of fit and predictability of models, respectively [17]. According to our PCA score plots, differences of metabolite composition between the wild-type and the *cir1* mutant could be explained by 0.74 of the *R^2^X* and 0.63 of the *Q* ^2^ values (Figure 1A). These results suggest that metabolite profiles are consistent within biological replicates and that deletion of *CIR1* resulted in distinct metabolic characteristics in the mutant cells.

### Discriminative Metabolites in the Strain Lacking *CIR1*

We subsequently searched for discriminative metabolites that contributed to the separation of the metabolite profiles of the *cir1* mutant from the wild-type. In order to discover them, student's *t*-test was conducted for annotated peaks by interpreting the first weight vector in the PLS-DA, assuming that metabolite concentrations follow a normal distribution in the same genetic background (Figure 1B). PLS-DA is commonly used to detect hidden variables that focuses on class separation [18]. Discriminative metabolites were selected using variable importance of the projection (VIP >1.0) and their *p*-value statistics (*p*<0.005).

Total 18 discriminative metabolites were identified in the *cir1* mutant, and their levels were significantly different from those of the wild-type (Table 1). To illucidate inter-individual variations in the wild-type and the *cir1* mutant according to iron availability, the yields of these metabolites were compared using box-and-whiskers plots (Figure 2). Most of these discriminative metabolites exhibited small changes when they were compared between low- and high-iron medium indicating that iron availability triggers only marginal differences in *C. neoformans* at least under the current experimental conditions used. The previous transcriptome analysis revealed that, in the wild-type strain, 483 genes were down-regulated and 250 genes were up-regulated more than 2-fold in low-iron versus high-iron medium based on Q-value statistics. The majority of differentially expressed genes in the wild-type strain upon iron availability included those related to iron transport and homeostasis, and DNA metabolism and repair. This and the results of our current metabolite profiling suggested that altered transcript levels in the wild-type strain did not produce a significant change in the metabolic flux in the wild-type strain. Our data also agreed with the recent study by Shakoury-Elizeh et al., who suggested that alterations in protein or transcript levels do not always correlate with the metabolic flux in *S. cerevisiae* because a metabolic pathway is largely determined by enzyme kinetics and substrate concentrations [11]. The influence of the gene *CIR1* on metabolism was dramatic in *C. neoformans*. Glycolytic pathway intermediates and ergosterol related compounds were increased in the *cir1* mutants while lactic acid, propylstearate, propylpalmitate, peiperidone and sucrose were decreased compared to the wild-type. Among the discriminative metabolites in the *cir1* mutant, the highest magnitude changes occurred in glucose, inositol, arabitol, ergosterol and ergostene.

### Cir1 Influences the Glycolytic Pathway and Respiration

The increase in glucose in the *cir1* mutant was of particular interest, as it implied that major carbon assimilation processes are affected by the deletion of *CIR1* in *C. neoformans*. Glucose is the main carbon source for energy production, is metabolized to pyruvate via glycolysis, and is oxidized via the tricarboxylic acid (TCA) cycle and respiration. It is known that iron influences both the TCA cycle and respiration. Two TCA cycle enzymes, aconitase and succinate dehydrogenase, contain Fe-S clusters, and complexes I–IV in the electron transport chain contain numerous Fe-S clusters as well as heme. In *S. cerevisiae*, expression of several genes involved in the TCA cycle and the electron transport chain were significantly down-regulated in cultures grown under iron deficient conditions [19]. Furthermore, recent metabolite analysis by Shakoury-Elizeh et al. revealed that iron deficiency caused depletion of glucose and glycolytic intermediates and increased flux of glucose through the glycolytic pathway for energy production but decreased respiration in *S. cerevisiae* [11]. Our metabolite analysis indicated that levels of glucose and glycolytic intermediate glycerol were increased in the *cir1* mutants. These results imply possible up-regulation of the glycolytic pathway and respiration in the *cir1* mutant, presumably due to increased intracellular iron levels, caused by deletion of *CIR1*. Indeed, a previous study showed that Cir1 acts not only as a transcriptional activator but also as a transcriptional repressor of genes required for iron uptake, which is also supported by the reduced growth phenotype of the mutant cells in the presence of excess iron or phleomycin, which induces DNA breakage via iron-catalyzed oxidation [4]. To confirm this hypothesis, we determined total cellular iron contents of the cells. The results suggested that total iron levels were significantly increased in the *cir1* mutant compared to the wild-type strain both in low- and high-iron conditions (Table 2). Taken together, these results show that deletion of *CIR1* in *C. neoformans* leads to increased intracellular iron levels and subsequent up-regulation of iron-requiring process like the glycolytic pathway and respiration. These results are opposite to what had been observed in iron-deficient *S. cerevisiae* cells. Therefore, we could suggest that flux of glucose might be reduced in *C. neoformans* due to deletion of *CIR1*, which resulted in increased glucose level in the *cir1* mutants.

### Cir1 Influences Ergosterol Biosynthesis

Levels of ergosterol and its derivatives were also increased in the *cir1* mutant (Figure 2). Ergosterol is the major fungal cell membrane constituent and is a target of the azole antifungal drugs. The biosynthesis pathway for ergosterol is highly conserved among fungi and the enzymes involved are encoded by approximately 22 genes [20]. Iron, as form of heme, is known to influence the synthesis of ergosterol because two enzymes, Erg5 and Erg11 encoding C-22 desaturase and C14α-methyl sterol demethylase respectively, belong to the heme containing cytochrome P450 protein family [21]. Moreover, Erg3 and Erg25 encoding Δ^5,6^ sterol desaturase and C-4 methyl sterol oxidase respectively contain non-heme iron.

In the current study, the effect of deletion of *CIR1* on ergosterol biosynthesis was further investigated by measuring expression of genes required for the pathway by Q-RT-PCR. These analyses suggested that in a high-iron medium, expression of *ERG11* (CNA00300) and *ERG24* (CNA01070) was up-regulated (2.55-fold and 2.70-fold, respectively) in the *cir1* mutant than in the wild-type strain (Figure 3). Moreover, in a low-iron medium, the expression of *ERG11* was even more significantly increased (4.25-fold) in the *cir1* mutant compared to the wild-type, which suggested that Cir1 implicates transcriptional regulation of *ERG11* and *ERG24*. The previous study showed that the *cir1* mutants are more resistant to miconazole, of which phenotype was supported by increased expression of genes encoding enzymes for ergosterol synthesis in transcriptome of the mutant cells [4]. For further confirmation of up-regulation of ergosterol biosynthesis in the *cir1* mutants, we evaluated the sensitivity of the mutant cells to various azole antifungal drugs including miconazole (Figure 4). Our data confirmed that the *cir1* mutants are more resistant to fluconazole, miconazole and ketoconazole than the wild-type.

Taken together, the results of our current metabolite analysis suggested that ergosterol synthesis was largely affected by deletion of *CIR1*, which caused alteration of sensitivity to azole antifungal drugs. Up-regulation of genes required for the pathway and increased cellular iron levels might contribute altered ergosterol metabolism in the *cir1* mutant. In addition to ergosterol itself, our metabolite analysis indicated that some of its derivatives were increased in the *cir1* mutants. Abundance of ergostene and methyl-ergostadiene were highly increased in the *cir1* mutants implying that composition of membrane sterol was changed significantly in the mutant cells. The same ergosterol derivatives were already shown to be associated with resistance to azole antifungal drug in *Candida albicans* [22]. Our data revealed that loss of Cir1 functions resulted in remodeling of ergosterol metabolism and membrane biosynthesis.

### Cir1 Regulates Inositol Metabolism

Inositol (myo-inositol) is an essential structural basis for many secondary messengers including inositol phosphates, phosphatidylinositol (PI) and phosphatidylinositol phosphate (PIP) lipids in a cell. Inositol can be synthesized from glucose 6-phosphate by 2 sequential processes: the first, conversion of glucose 6-phosphate to inositol 3-phosphate, is mediated by inositol-3-phosphate synthase, and the second, dephosphorylation of inositol 3-phosphate to generate inositol, by inositol monophosphatase. Cells can also import inositol from the environment via inositol transporters [23]. Several studies have shown that inositol and its derivatives play an important role in signaling pathways, sexual reproduction and virulence of the fungus [24], [25], [26], [27]. Involvement of inositol in pathogenesis of *C. neoformans* has been of particular interest since its elevated levels was found in the mammalian brain, which is the most preferred host niche of the fungus [26], [28], [29], [30]. The genome of *C. neoformans* contains an unusually large gene family encoding at least 10 inositol transporters [26], and the recent study by Wang et al. suggested that, among them, *ITR1A* and *ITR3C* are the major inositol transporters and are required for full virulence of *C. neoformans* [31]. The contribution of inositol metabolism to virulence has also been described in *Candida albicans*, which can acquire inositol by *de novo* synthesis or by importing it; either mechanism is sufficient to support the wild-type growth *in vitro* and full virulence [32]. More importantly, enzymes associated with inositol metabolism, such as phospholipases and lipases, contribute significantly to virulence of a number of other fungal pathogens [33].

We found that the level of inositol was significantly increased in the *cir1* mutants than in the wild-type strains, suggesting that inositol metabolism is up-regulated by deletion of *CIR1*. This observation led us to compare the levels of expression of genes in inositol phosphate metabolism in the *cir1* mutant and to compare with that in the wild-type. Several genes in the pathway were selected based on the previous transcriptome data and their relative expression levels were investigated. Figure 5 shows the results of Q-RT-PCR for inositol oxygenases (CNG03010 and CNH03170), myoinositol oxygenase (CNF03950), and methylmalonate-semialdehyde dehydrogenase (CND02030) in the pathway. Overall expression levels of these genes, except myoinositol oxygenase, were significantly increased in the *cir1* mutant. These results suggested that inositol metabolism is indeed up-regulated in the *cir1* mutant, which could explain why the metabolite analysis resulted in increased inositol contents in the mutant cells. We should also note here that the overall trends of expression of the genes were similar to previous transcriptome analysis [4]. Taken together, results of the current study suggested Cir1 influences on inositol metabolisms via transcriptional regulation of key genes in the pathway. In summary, our metabolome analysis revealed that deletion of the gene encoding a major iron regulatory transcription factor Cir1 largely influences iron required metabolisms such as the glycolytic pathway and respiration as well as ergosterol biosynthesis. Moreover, inositol metabolism is regulated by Cir1 in *C. neoformans*.

## Supporting Information

Table S1 Primers used for quantitative real-time RT-PCR.
(DOCX)

Table S2 The summarized metabolite profiles from GC-MS analysis.
(XLSX)
