# Supplementary material for: Influence of Iron Regulation on the Metabolome of Cryptococcus neoformans
Source: PLoS One. 2012 Jul 23;7(7):e41654. doi: 10.1371/journal.pone.0041654 (PMC3402442; doi:10.1371/journal.pone.0041654)
Supplement: Table S1 — Primers used for quantitative real-time RT-PCR. (DOCX) [file pone.0041654.s001.docx]

**Supplementary materials**

Table S1. Primers used for quantitative real-time RT-PCR

| Gene ID | | Primer sequence |  |
| --- | --- | --- | --- |
| CNJ03110 | Forward | AGAGGGCTGGTGAGCTCAAC | 18S |
|  | Reverse | TCTGCATGATGGTGACGATTC |  |
| CNA00300 | Forward | TCCCCCAGGTCCAGCAA |  |
|  | Reverse | AACCACGGTGGGAAAAATTG |  |
| CNA01070 | Forward | TTTGCCTGCAACGATGCA |  |
|  | Reverse | GCATCTTTCCACCCCTGTACA |  |
| CNA05010 | Forward | CCACGATGGCGATATGATCA |  |
|  | Reverse | GGGCGGGACCGTTGA |  |
| CNF03950 | Forward | GGCAAGCCCGAGTGGAT |  |
|  | Reverse | AGAGAAGCTTGCCGAGATCGT |  |
| CNG03010 | Forward | AAGGATGGGAAGCCGGAAT |  |
|  | Reverse | CTTGCCGAGGTCGTGGAT |  |
| CNH03170 | Forward | GCGCTACAATGGGCGTTT |  |
|  | Reverse | TTGCGTCGACAAGGGTGTT |  |
| CND02030 | Forward | CGAGCCACTTGGTGTTGGA |  |
|  | Reverse | AAAGGTATCATAGCCGGGAAGTT |  |
